# Structures of the *Mycobacterium tuberculosis* GlpX protein (class II fructose-1,6-bisphosphatase): implications for the active oligomeric state, catalytic mechanism and citrate inhibition

**DOI:** 10.1107/S2059798318002838

**Published:** 2018-04-03

**Authors:** Nina M. Wolf, Hiten J. Gutka, Farahnaz Movahedzadeh, Celerino Abad-Zapatero

**Affiliations:** a Institute for Tuberculosis Research, University of Illinois at Chicago, Chicago, Illinois, USA; b Department of Medicinal Chemistry and Pharmacognosy, University of Illinois at Chicago, Chicago, Illinois, USA; c Oncobiologics Inc., Cranbury, New Jersey, USA; d Center for Biomolecular Sciences, University of Illinois at Chicago, Chicago, Illinois, USA

**Keywords:** class II fructose-1,6-bisphosphatase, *Mycobacterium tuberculosis*, TCA cycle intermediates, GlpX, allosteric regulation

## Abstract

The crystal structures of native class II fructose-1,6-bisphosphatase (FBPaseII) from *Mycobacterium tuberculosis* at 2.6 Å resolution and two active-site protein variants are presented. The variants were complexed with the reaction product fructose 6-phosphate (F6P). The Thr84Ala mutant is inactive, while the Thr84Ser mutant has a lower catalytic activity. The structures reveal the presence of a 222 tetramer, similar to those described for fructose-1,6/sedoheptulose-1,7-bisphosphatase from *Synechocystis* (strain 6803) as well as the equivalent enzyme from *Thermosynechococcus elongatus*. This homo­tetramer corresponds to a homologous oligomer that is present but not described in the crystal structure of FBPaseII from *Escherichia coli* and is probably conserved in all FBPaseIIs. The constellation of amino-acid residues in the active site of FBPaseII from *M. tuberculosis* (*Mt*FBPaseII) is conserved and is analogous to that described previously for the *E. coli* enzyme. Moreover, the structure of the active site of the partially active (Thr84Ser) variant and the analysis of the kinetics are consistent with the previously proposed catalytic mechanism. The presence of metabolites in the crystallization medium (for example citrate and malonate) and in the corresponding crystal structures of *Mt*FBPaseII, combined with their observed inhibitory effect, could suggest the existence of an uncharacterized inhibition of this class of enzymes besides the allosteric inhibition by adenosine monophosphate observed for the *Synechocystis* enzyme. The structural and functional insights derived from the structure of *Mt*FBPaseII will provide critical information for the design of lead inhibitors, which will be used to validate this target for future chemical intervention.

## Abstract 2

Structures of native and variants (T84S and T84A) of *M. tuberculosis* class II fructose-1,6-bisphosphatase are presented and compared with those of other homologs. The structure is a 222-symmetric homotetramer. Citrate was bound at a dimer interface and was found to be an inhibitor.

## 1. Introduction   

Tuberculosis (TB) affects two billion individuals, with ten million new cases and 1.5 million deaths reported each year (Quan *et al.*, 2017 ▸). Multidrug-resistant, extensively drug-resistant and totally drug-resistant strains of tuberculosis complicate the already tedious treatment protocol (Balganesh *et al.*, 2008 ▸). The World Health Organization has the goal of eliminating the disease by 2050 (Koul *et al.*, 2011 ▸). To accomplish this feat, drugs with new modes of action need to be explored.

In the past, the search for new TB drugs has primarily been through whole-cell screening methodologies. Few strategies have used a target-driven methodology. A target-driven study for isocitrate lyase (ICL) from the glyoxylate-shunt pathway was not pursued (Koul *et al.*, 2011 ▸) owing to pleiotropic effects in ICL-deficient *Mycobacterium tuberculosis* (*Mtb*). A good target must be an enzyme that is essential for the survival of the bacterium and that has an active site or regulatory pocket that can be blocked selectively by suitable chemical entities. The main advantages of a target-based approach are a known mechanism of action and the possibility of expedient optimization of the affinity and physicochemical properties of the initial leads by structure-based drug design. However, the target needs to be validated by genetic as well as chemical methods. This requires the early identification of lead compounds that are efficacious in cell assays and that are selective towards the target of interest. Because of the paucity of target-based strategies, we have undertaken a target-driven approach for an essential enzyme in the gluconeogenesis pathway of *Mtb*.

Gluconeogenesis is critical for *Mtb* infection and late persistence owing to the need for a primary carbon source (*i.e.* glycerol and fatty acids) during this phase (Marrero *et al.*, 2010 ▸). Phosphoenolpyruvate carboxykinase (PEPCK) catalyzes the first committed step of gluconeogenesis and has been shown to be important for *Mtb* growth during active and latent stages of the disease (Marrero *et al.*, 2010 ▸). In addition, three enzymes involved in glycerol metabolism have also been considered as possible targets: triosephosphate isomerase (TPI; Trujillo *et al.*, 2014 ▸), fructose bisphosphate aldolase (FBA; Puckett *et al.*, 2014 ▸) and fructose-1,6-bisphosphatase (FBPase; Gutka *et al.*, 2015 ▸). *Mtb* fructose-1,6-bisphosphatase (*Mt*FBPaseII), the enzyme encoded by the *glpX* gene, is the third component of the *glpFKX* operon involved in the growth of *Mtb* using glycerol as the sole carbon source (Donahue *et al.*, 2000 ▸). *Mt*FBPaseII catalyzes the only unidirectional step in the gluconeogenesis pathway of the pathogen (Ganapathy *et al.*, 2015 ▸).

Two recent studies using genetic knockout and complementation protocols have shown that gluconeogenesis and the bisphosphatase activity of *Mt*FBPaseII are essential for virulence in *Mtb* grown using glycerol as an alternative carbon source (Gutka *et al.*, 2015 ▸; Ganapathy *et al.*, 2015 ▸). In addition, extended (180 d) monitoring of the survival profile of mice infected with Δ*glpX* mutant *Mtb* showed that the gene is essential for growth in the acute phase and for long-term survival during the chronic phase of *Mtb* infection (Gutka *et al.*, 2015 ▸). The FBPase encoded by the *glpX* gene has also been validated as an essential target in the closely related species *M. marinum* (Tong *et al.*, 2016 ▸) and in the important bacterial pathogen *Francisella tularensis* (Brissac *et al.*, 2015 ▸).

FBPase enzymes have been assigned to five classes (I–V) based on their amino-acid sequences. *Mt*FBPaseII comprises the only FBPase activity in *Mtb* (Movahedzadeh *et al.*, 2004 ▸) and is closely related to the class II FBPase from *Escherichia coli* (Brown *et al.*, 2009 ▸). Eukaryotes primarily use class I enzymes, which are the most widely distributed of the classes, but prokaryotes can contain all five classes. Class IV enzymes, bifunctional FBPase/inositol monophosphatases, are unique to archaea, while class V enzymes are unique to thermophilic prokaryotes (Brown *et al.*, 2009 ▸). Many of these enzymes are sensitive to lithium, including those from *Mtb* (Bondoc *et al.*, 2017 ▸), *E. coli* (Brown *et al.*, 2009 ▸) and the dual-function fructose-1,6/seudoheptulose-1,7-bisphosphatase (FBP/SBPase) from *Thermosynecho­coccus elongatus* (Cotton *et al.*, 2015 ▸). FBPases often require divalent metals such as Mg^2+^, Mn^2+^ or Zn^2+^ for activity.

The structures of FBPases from the five different classes consist of two α/β domains, one with two layers (α–β; CATH code 3.30.540) adjacent to another domain with three layers (α–β–α; CATH code 3.40.190), forming a five-layered sandwich (α–β–α–β–α). However, although the resulting β-sheets in the two domains are approximately orthogonal to each other, the number of strands in each sheet, the boundaries of the strands and the topology connecting the β-strands varies in the different classes. The composition and topology of the two main β-sheets in classes I and II have been summarized and are illustrated in Supplementary Tables S1(*a*)–S1(*c*) and Supplementary Figs. S1(*a*)–S1(*c*). Historically, the most extensively studied FBPases are the members of class I. The mammalian phosphatase (*e.g.* pig kidney) representative of this class is tetrameric (222 symmetry) and is allosterically regulated by a conserved adenosine monophosphate (AMP) site found in the amino-terminal region of the polypeptide chain. The activity of the *E. coli* class I FBPase (*Ec*FBPaseI) is also regulated by AMP, citrate and phosphoenolpyruvate (PEP), resulting in inhibition or activation depending on the different concentrations of these effectors (Hines *et al.*, 2007 ▸).

The structures of three enzymes from the class II FBPases have been reported and are available from the Protein Data Bank (PDB). The first structurally characterized member of the class II FBPases was that from *E. coli* (*Ec*FBPaseII), which has been reported in various forms (Brown *et al.*, 2009 ▸): the apo enzyme (PDB entry 3big), a complex with inorganic phosphate (PDB entry 3bih) and a complex with the substrate fructose 1,6-bisphosphate (FBP; PDB entry 3d1r). A second class II FBPase, FBP/SBPase from the cyanobacterium *Synecho­cystis* strain 6803, has also been described (Feng *et al.*, 2014 ▸; PDB entries 3roj and 3rpl) and, most recently, the same dual-function enzyme from *T. elongatus* has been reported with sedoheptulose-7-phosphate bound (PDB entry 5a5l; Cotton *et al.*, 2015 ▸).

This work describes the crystallization and structure refinement of wild-type *Mt*FBPaseII in its apo form at 2.6 Å resolution. In addition, we present structures of F6P-bound forms for two variants of the active-site residue Thr84: an inactive (T84A) variant (2.3 Å resolution) and a partially active (T84S) variant (2.2 Å resolution), the enzymatic properties of which have been described previously (Bondoc *et al.*, 2017 ▸). We discuss the similarities and the differences between the *E. coli* FBPases (*Ec*FBPaseI and *Ec*FBPaseII) and *Mt*­FBPaseII and provide conclusive evidence that *Mt*FBPase is a homotetramer. The structure of this class II FBPase is compared with structures within the same class, namely those from *Synechocystis* and *T. elongatus*, which are found to be more structurally similar to *Mt*FBPaseII than to *Ec*­FBPaseII.

The analysis and comparison of the structures of the two active-site variants provides support for the catalytic mechanism suggested for the *E. coli* enzyme. The three refined structures of *Mt*FBPaseII revealed the presence of citrate and malonate, which were present in the crystallization medium. Inhibition by these metabolites was also observed in enzymatic assays and could be relevant for the regulation of this class of enzymes. These results provide structural, biochemical and functional data for the future identification and design of specific lead inhibitors that will be used to validate *Mt*­FBPaseII as an effective target for chemical intervention.

## 2. Materials and methods   

### 2.1. Protein purification and crystallization   

Wild-type apo *Mt*FBPaseII was prepared as described previously (Bondoc *et al.*, 2017 ▸). After initial purification using N-terminal His-tag affinity chromatography, the His-tagged protein was further purified using a Superdex 200 HiLoad 26/60 column (GE Healthcare Biosciences, USA) on an ÄKTApurifier FPLC system (GE Healthcare Biosciences, USA) pre-equilibrated with 20 m*M* tricine pH 8.0, 50 m*M* KCl, 1 m*M* MgCl_2_, 15% glycerol buffer. A flow rate of 1 ml min^−1^ was used for operation, and the purity of the fractions was assessed by SDS–PAGE. The protein was concentrated to 10 mg ml^−1^. All protein-purification steps were performed at 25°C. The protein concentration was estimated from the absorbance at 280 nm with a NanoDrop spectrophotometer (Thermo Fisher Scientific, Waltham, Massachusetts, USA) using an extinction coefficient of 17 420 *M* ^−1^ cm^−1^ (Bondoc *et al.*, 2017 ▸). The T84S and T84A variants were prepared in a similar manner without size-exclusion chromatography.

Crystallization trials were performed by the hanging-drop vapor-diffusion method using crystallization screens (Index, Crystal Screen, PEGRx, SaltRx and PEG/Ion) from Hampton Research, Aliso Viejo, California, USA. For the F6P complexes, a sodium malonate screen was used to optimize the crystal size and quality. A 1:1 ratio of protein to precipitant was used (Gutka, Franzblau * et al.*, 2011 ▸) and the crystals grew at 25°C. Apo *Mt*FBPaseII crystals were obtained using a reservoir solution consisting of 1.0 *M* ammonium citrate tribasic pH 7.0, 1% PEG 3350. *Mt*FBPaseII–F6P complex crystals were obtained by incubating *Mt*FPBaseII with 1 m*M* F6P or FBP for 30 min at room temperature and then mixing the solution with reservoir solution consisting of 2.4 *M* sodium malonate pH 6.0.

### 2.2. Data collection, structure solution and refinement   

Crystals for synchrotron data collection were cooled in liquid nitrogen after soaking with cryoprotectant solution consisting of the reservoir solution plus 20% glycerol. X-ray diffraction data for the three structures were collected from a single cooled crystal using a MAR300 CCD detector on the Life Sciences Collaborative Access Team (LS-CAT) 21-ID-G beamline at the Advanced Photon Source, Argonne National Laboratory, Illinois, USA. For the apo *Mt*FBPaseII crystal, the crystal-to-detector distance was set to 350 mm and 360 frames were collected with a 0.5° scan width and a 6.7 s exposure time. For the T84S *Mt*FBPaseII–F6P crystal, the crystal-to-detector distance was set to 300 mm and 360 frames were collected with a 0.5° scan width and a 1.6 s exposure time. For the T84A *Mt*FBPaseI–F6P crystal, the crystal-to-detector distance was set to 280 mm and 200 frames were collected with a 1° scan width and 7.3 s exposure time.

The diffraction data were integrated, reduced, processed and scaled with *HKL*-2000 (Otwinowski & Minor, 1997 ▸). The structure solutions for all crystal forms were obtained by molecular replacement using a partially refined structure of the apo enzyme as a model. This initial structure was refined (*R*_work_ = 0.329, *R*_free_ = 0.390) against an earlier data set that had also been solved by molecular replacement (Gutka, Franzblau *et al.*, 2011 ▸) with *MOLREP* (Vagin & Teplyakov, 1997 ▸, 2010 ▸) using the *E. coli* enzyme (PDB entry 3d1r) as the initial search model.

The crystal structures of apo *Mt*FBPaseII, the T84A *Mt*FBPase–F6P complex and the T84S *Mt*FBPase–F6P complex were solved by molecular replacement and refined to 2.6, 2.3 and 2.2 Å resolution, respectively, in space group *P*6_1_22. All structures were in the same crystal form with a noncrystallographic symmetry dimer in the asymmetric unit. Although varying slightly in unit-cell dimensions, the three crystal structures were isomorphous. The apo enzyme crystals were well formed hexagonal prisms ranging in size from 60 to 100 µm in the long axis. The crystals of the complex also had a hexagonal cross-section, but the vertices and edges were rounder.

*Coot* was used for minor model revision and refinement as well as for rebuilding the exposed helical residues corresponding to Arg235–Glu258 of *Ec*FPBaseII; this region is significantly shorter in *Mt*FBPaseII because of a deletion of 13 residues. Ligand fitting, placement of water and building and refinement of additional crystallization molecules from the medium (glycerol, citrate and malonate) were also performed using *Coot*. The presence of the larger ligands (F6P, citrate and malonate) in the crystal structures was validated with polder maps during model building and refinement (Liebschner *et al.*, 2017 ▸) using *PHENIX* (v.1.12-2829). The final refinement *R*_work_ and *R*_free_ were 0.211 and 0.265 for the apo enzyme (PDB entry 6ayy), 0.190 and 0.247 for the T84A mutant (PDB entry 6ayv), and 0.224 and 0.258 for the T84S mutant (PDB entry 6ayu), respectively. Full data-collection and refinement statistics are given in Table 1 ▸.

### 2.3. Enzymatic analysis and compound affinity   

The FBPase activity of wild-type *Mt*FBPaseII was measured using the malachite green assay (Bondoc *et al.*, 2017 ▸) in the presence of α-ketoglutarate, citrate, isocitrate, malate, malonate and oxaloacetate. Compounds were tested in triplicate at concentrations of 5000, 3000, 2500, 1300, 630, 310 and 160 µ*M* with positive (no compound) and negative (no enzyme) controls in parallel. Fitting was performed with *GraphPad Prism* (v.7.0b; GraphPad Software, La Jolla, California, USA). The coupled assay (Bondoc *et al.*, 2017 ▸) was used to obtain kinetic data, which were fitted to a nonlinear Michaelis–Menten equation.

## 3. Results   

### 3.1. Structure of the *Mt*FBPase monomer   

The *Mt*FBPaseII monomer is a compact, globular protein with two distinct α/β-type domains (I and II) arranged in an alternating multilayer fashion to form an α/β/α/β/α-fold five-layer sandwich as described above. Secondary-structure analysis of the *Mt*FBPaseII monomer reveals that there are 12 α-helices and 14 β-strands connected by loops of varying lengths (Supplementary Fig. S2).

The structures of the three *Mt*FBPaseII monomers are very similar to the class II structures discussed above, with the highest similarity being between the T84S variant and PDB entry 3rpl, with an r.m.s.d. of 0.762 Å; the T84S variant is also the most distant from PDB entry 3d1r, with an r.m.s.d. of 0.929 Å (Table 2 ▸). The two variant *Mt*FBPaseII structures differ with an r.m.s.d. of 0.265 Å; the r.m.s.d. between each variant and the apo structure is less than 0.4 Å. *Mt*FBPaseII, like *Ec*FBPaseII, also lacks the insertion at residues 76–89 in PDB entry 3rpl. Only *Mt*FBPaseII lacks the insertion at residues 237–248 in PDB entry 3d1r. The C-terminus is disordered after the last β-strand for the structures with PDB codes 5a5l and 3d1r, while an unstructured loop continues on for 10–13 residues in chain *A* of *Mt*­FPBaseII and PDB entry 3rpl, forming the allosteric AMP site in the *Synechocystis* enzyme. Structural alignment of the four FBPaseII enzymes documents strong conservation among FBPases from different species (Supplementary Fig. S3).

**Figure 1:**
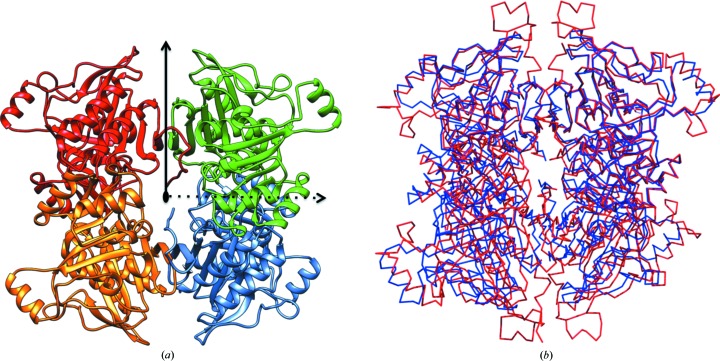
222 homotetramer of *Mt*FBPaseII. (*a*) The T84S *Mt*FBPaseII tetramer as calculated by *PISA* (http://www.ebi.ac.uk/pdbe/prot_int/pistart.html; Krissinel & Henrick, 2007 ▸) is displayed with each of the four subunits in a different color. The three orthogonal dyads are indicated: the noncrystallographic horizontal dyad is indicated by a dashed line, the crystallographic dyad described in *Ec*FBPase II (PDB entry 3drl) is indicated by a vertical solid line and the third dyad, perpendicular to the other two, is marked by the black oval in the center. (*b*) C^α^ trace of the T84S *Mt*FBPaseII tetramer (blue) superimposed with that of the FBP/SBPase tetramer from *Synechocystis* (PDB entry 3rpl; red). Alignment of *Mt*FBPaseII with PDB entry 3rpl was performed by superposing the C1 and C2 dimers. Differences can be seen in the length of the C-terminus and the extensions at the top and bottom of the figure. Subunits are identified as C1–C4 beginning at the top left and continue clockwise. Figures were prepared with *UCSF Chimera*.

The monomeric structure is very similar to the *Ec*FBPaseII structure used as a search model for molecular replacement, except for a marked difference in the α-helix 9 (H9) region of the parent structure. *Mt*FBPaseII lacks about 13 residues that constitute part of the long H9 and the preceding five-residue loop in the *Ec*FBPaseII structure. As a result, the corresponding H9 in the *Mt*FBPaseII structure is much shorter and the overall loop–H9–loop region (Arg230–Tyr243) does not protrude as far away from the tetramer core structure (Fig. 1 ▸).

### 3.2. Comparison of class I *versus* class II FBPases   

FBP/SBPase from the cyanobacterium *Synechocystis* (Feng *et al.*, 2014 ▸; PDB entry 3rpl) is a unique dual-function member of the class II FBPase family which catalyzes two separate reactions in the Calvin cycle. The amino-acid sequence shares low (<10%) identity with the mammalian class I phosphatases, but shares a more significant sequence identity of 39% (57% similarity) with *Ec*FBPaseII (PDB entry 3d1r). Structural comparison of chain *A* of these two proteins yielded an r.m.s.d. of 0.997 Å. This is an r.m.s.d. difference that is comparable to the largest difference observed in Table 2 ▸ (1.014 Å) between the homologous dual-function enzyme from *T. elongatus* (PDB entry 5a5l) and the *Ec*FBPaseII enzyme. The crystal structure (PDB entry 3rpl) also revealed a globular shape and a similar five-layered (α–β–α–β–α) sandwich. The two α/β domains are topologically analogous in the number of β-strands (β1–β14) and in the way in which the two orthogonal β-sheets relate to each other. Although the class I and class II FBPases share the same five-layered structure, the boundaries of the β-strands and the topology of the two β-sheets (A and B) are different (Supplementary Tables S1*a*–S1*c*).

The allosteric effector for the dual-enzyme FBP/SBPase from the cyanobacterium (AMP) is found in an extended C-terminal extension at the center of the full tetramer in the asymmetric unit. The association and contact interfaces of the four chains comprising the 222 tetramer differ from the tetramer characterized for the mammalian class I phosphatases, as illustrated by Feng *et al.* (2014 ▸). The main differences between the *E. coli* and *Synechocystis* structures are (i) near residues 76–89, where PDB entry 3rpl has an insertion including a disulfide bond, (ii) residues 247–259, where PDB entry 3rpl includes three β-turns while PDB entry 3d1r contains a one-turn helix followed by a loop, and (iii) the C-terminus, which is longer in PDB entry 3rpl (Supplementary Fig. S3). Of note is the presence of a disulfide bond connecting residues Cys75 and Cys99 in PDB entry 3rpl. It has been suggested that the enzyme is found to be inactive as a dimer and is activated by the addition of dithiothreitol (DTT) to break the disulfide bond and allow the formation of an active tetramer (Weeks *et al.*, 1999 ▸).

The structure of the *Synechocystis* enzyme is very similar to that of the class II FBP/SBPase from *T. elongatus* (PDB entry 5a5l; Cotton *et al.*, 2015 ▸), with an r.m.s.d. of 0.559 Å for 96% of the possible C^α^ pairs (325/338) for the *A* chains, resulting in essentially the same structure except for some minor differences at residues 248–260. The sequence identity between the two structures is 77% (85% similarity; Supplementary Fig. S3). The last ten residues at the C-terminus, where AMP is bound in the cyanobacterial structure (PDB entry 3rpl), are not structurally resolved in the *T. elongatus* class II FBP/SBPase (Cotton *et al.*, 2015 ▸).

The active site of PDB entry 5a5l has a phosphate group near a molecule of sedoheptulose-7-phosphate. The high degree of structure conservation in the residues in the active site has been documented, supporting the proposed catalytic mechanism (Brown *et al.*, 2009 ▸). The possibility of redox regulation of the *Synechocystis* enzyme structure owing to the inactivity of the enzyme without DTT has been suggested (Feng *et al.*, 2014 ▸). However, no evidence for the existence of a disulfide bond between Cys75 and Cys99 was found and significant conformational rearrangement would be required for them to come into contact (Cotton *et al.*, 2015 ▸).

### 3.3. Structure of the tetrameric oligomer and its relation to other tetrameric FBPases   

The initial crystallographic characterization of the crystals of apo *Mt*FBPaseII (Gutka, Franzblau *et al.*, 2011 ▸) revealed the presence of a noncrystallographic dimer that differs from that suggested to be the functional unit of the *E. coli* enzyme structure (PDB entry 3d1r). Further analysis indicated that the noncrystallographic dyad was inclined approximately 55° from the 6_1_ screw axis.

Analysis of the packing and subunit interactions obtained from *PISA* for the three structures and the size-exclusion chromatography (SEC) data for molecular-weight estimation (Gutka, Rukseree * et al.*, 2011 ▸) provides strong evidence that *Mt*FBPase is a functional tetramer. The PDB structures of *Ec*FBPaseII (PDB entries 1ni9, 3bih, 3big and 3d1r) all only contain a monomer of 36 kDa in the asymmetric unit, but the symmetry-element constraints of the space group (*P*422) indicate the presence of a 222 tetramer at the 222 positions in the crystal cell by duplication of the elongated dimer (Brown *et al.*, 2009 ▸).

The two subunits in the asymmetric unit have closely interacting residues at the interface, with the residues of α-helix 3 interacting with those of β7. The residues of α-helix 6 also interact with loop residues between strands β11 and β12. These dimer interface interactions are different from the antiparallel β-strand–β-strand interactions between the two subunits observed in *Ec*FBPaseII (Brown *et al.*, 2009 ▸). Such β-strand–β-strand interactions are also observed in the overall *Mt*FBPaseII tetramer as predicted by *PISA* (Fig. 1 ▸). *PISA* predicted the T84S variant complexed with F6P to be stable as a tetramer with a free energy of dissociation (*ΔG* ^diss^) of 0.6 kcal mol^−1^ or as a dimer with a Δ*G* ^diss^ of 2.6 kcal mol^−1^. Similar results were found for T84A, with a tetramer assembly just below the acceptable Δ*G* ^diss^ positive threshold at −0.6 kcal mol^−1^ and a value of 1.5 kcal mol^−1^ for the dimer. However, apo *Mt*FBPaseII was not found to have a stable tetrameric structure, as the subunits appear to be associated less tightly. This observation may suggest that the binding of substrate/product could alter the inter-subunit association of the 222 tetramer.

Despite having the same overall architecture, the *Mt*FBPaseII monomer is very different from that of *Ec*­FBPaseI and also from the conventional class I FBPases represented by the extensively studied mammalian phosphatase from pig liver, as illustrated in Supplementary Tables S1(*a*)–S1(*c*) and Figs. S1(*a*)–S1(*c*). These differences are also reflected in the mode of association of both enzymes to form functional tetramers that are allosterically regulated in class I phosphatases (both mammalian and *E. coli*) and in the characterized class II FBPase from *Synechocystis*, which is allo­sterically regulated by AMP (Feng *et al.*, 2014 ▸).

### 3.4. Structure of the *Mt*FBPase–F6P complex   

**Figure 2:**
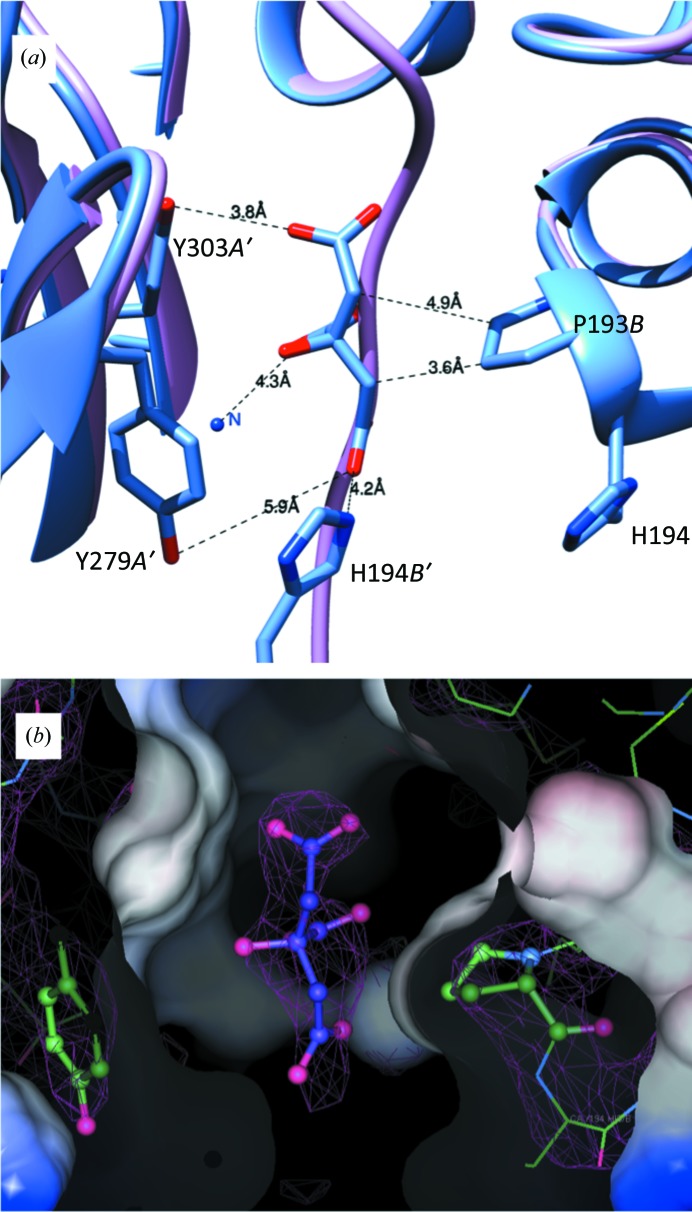
Citrate binding in the apo *Mt*FBPaseII structure. (*a*) The C-terminus of the T84S *Mt*FBPaseII structure (light purple) is displaced by a citrate molecule in the apo structure (blue). Distances to the most relevant contacts are annotated. The chains are denoted *A*′ (crystallographic symmetry-related chain *A*), *B* and *B*′. (*b*) The surface contacts of the citrate in the pocket and the corresponding electron density of the 2*F*_o_ − *F*_c_ map at 1.0σ are shown. The figures were prepared with *Coot*.

Binding of the product and the presence of malonate caused minor conformational changes in *Mt*FBPaseII. The largest movements are seen in the length of the C-terminus that is visible in the structure and in the region of residues 180–200 (shown in a black box in Supplementary Fig. S4). The C-terminus is the most mobile part of the structure, which may have implications for regulation, since this part is ordered and forms the binding pocket for AMP in the *Synechocystis* enzyme. Residues 180–200 are part of α-helix 10 (H10), which is located in the middle of a β–α–β sandwich. H10 contains the active-site residue Asp182. The T84S variant structure has the most visible residues in the structure, ten more than the apo structure. In the apo structure, the C-terminus is displaced by a citrate molecule from the crystallization medium (Fig. 2 ▸). In the two variant *Mt*FBPaseII–F6P complexes (T84A and T84S) the C-terminus is ordered (although with high *B* factors), with a malonate molecule from the medium found bound in the center of the tetramer at the interface of two monomers, occupying a position near the location of the AMP allosteric site in the *Synechocystis* enzyme (Fig. 5).

### 3.5. Active site   

**Figure 3:**
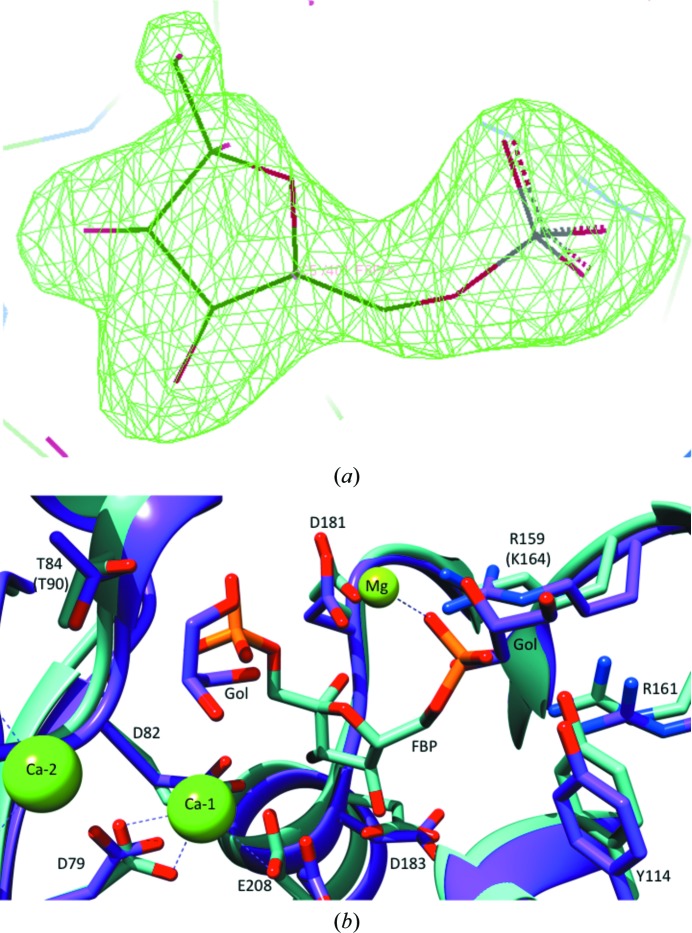
Active-site view of *Mt*FBPaseII with FBP. (*a*) The electron-density polder map of F6P indicates strong evidence for the presence of the product in the active site of T84S *Mt*FBPaseII. The figure was prepared with *Coot*. (*b*) The structure of apo *Mt*FBPaseII (purple) was superimposed with the structure of the homolog *Ec*FBPaseII (teal) in complex with the substrate FBP. The hydroxyl of Thr84 in wild-type *Mt*FBPaseII and the corresponding hydroxyl in T84S *Mt*FBPaseII superimpose at the same position. The hydrogen bond from Thr90 of *Ec*FBPaseII (corresponding to Thr84 in *Mt*FBPaseII) to FBP (teal) is 3.5 Å in length; Thr84 of *Mt*FPBaseII (purple) is within a 3.8 Å distance of the phosphate group. Gol represents a glycerol molecule. Ca^2+^ ions are only present in the *Ec*FBPaseII structure, while Mg^2+^ is present in both structures.

The crystals of the complexes with F6P diffracted to higher resolutions (2.3 and 2.2 Å, respectively; Table 1 ▸). Although the T84S mutant was co-crystallized in the presence of the substrate (FBP), only the product (F6P) was found in the active site, demonstrating that the enzyme is still active, as has previously been observed (Bondoc *et al.*, 2017 ▸). Superposition of the *Ec*FBPaseII structure (PDB entry 3d1r) with the *Mt*FBPaseII structures identifies the position of the missing phosphate group (Fig. 3 ▸). One Mg^2+^ ion is found in T84S *Mt*FBPaseII, coordinated to a glycerol molecule and to Asp79, Asp82 and Glu208 near the cleaved 1-phosphate group of the substrate. In the *Ec*FBPaseII structure, three ions are found in the active site: Ca^2+^-1 in the same position as the Mg^2+^ in *Mt*FBPase coordinated to three waters, Asp85 and Glu213; a second Ca^2+^ ion coordinated to four waters, Asp33 and Glu57; and an Mg^2+^ ion coordinated to five waters and the 6-phosphate (Fig. 3 ▸).

Subtle differences in the active site are seen, including a rotated orientation of Thr84. Thr84 is oriented towards the FBP in *Ec*FBPaseII and away from the F6P in *Mt*FBPaseII (Fig. 3 ▸). Although the serine-variant enzyme has significantly reduced activity (Bondoc *et al.*, 2017 ▸), the hydroxyl groups of the wild-type and T84S *Mt*FBPaseII structures superimpose at approximately the same position. The hydrogen bond from the hydroxyl group of Thr90 of *Ec*FBPaseII (Thr84 in *Mt*­FBPaseII) to FBP (teal) is 3.5 Å in length, while the hydroxyl group of Thr84 in *Mt*FPBaseII (purple) is just outside the standard hydrogen-bonding distance at 3.8 Å from the phosphate group (Fig. 3 ▸).

### 3.6. Implications for the mechanism   

The proposed mechanism involves two metal ions near the cleavable phosphate: one to stabilize the negative charge on the leaving group and one to coordinate the nucleophilic water (Brown *et al.*, 2009 ▸). The apo enzyme does not contain Mg^2+^ in the active site; instead Mg^2+^ is found in helices H8 and H9 in one chain, coordinated to Leu165 N, Gln164 N and Arg161 O and possibly stabilizing this loop. The F6P-bound structures contain one magnesium ion in a position that is proposed to bind FBP, coordinate the 1-phosphate and stabilize the transition state (Brown *et al.*, 2009 ▸). This magnesium ion is coordinated to Asp79 OD2, Glu208 OE2, Asp82 OD1, F6P O1 and glycerol. The positions of the hydroxyl group of Thr84 in the wild type and the corresponding hydroxyl in the T84S mutant are the same. However, the hydroxyl is reversed compared with that of the threonine residue in the complex of *Ec*FBPaseII with FBP.

### 3.7. Citrate inhibition   

The presence of citrate (in apo *Mt*FBPaseII) and malonate (in the T84S and T84A variants complexed with F6P) in the crystallization medium and in the resulting structures suggested the possibility of a weak affinity of some di/tricarboxylic acids towards the *Mt*FBPaseII enzyme. The presence of these molecules in the crystal structures was confirmed by calculating polder maps, resulting in correlation coefficients [peak CC(1,3)] of 0.86 for citrate in apo *Mt*FBPasII and 0.84 and 0.89 for malonate in the structures of T84S and T84A *Mt*FBPaseII, respectively.

**Figure 4:**
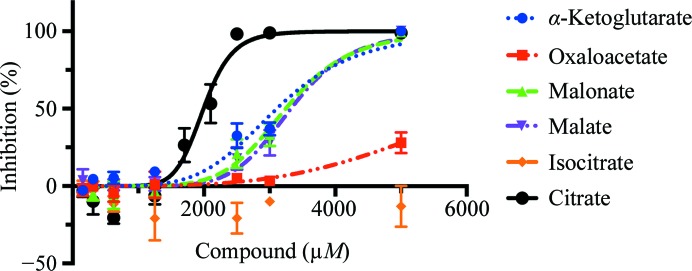
Inhibition of *Mt*FBPase by different di/tricarboxylic acids. Inhibition curves of *Mt*FBPase against various compounds related to the TCA cycle are shown. All were found to have IC_50_ values greater than 2 m*M*, with citrate having the strongest affinity.

Several tricarboxylic acid (TCA) cycle intermediates were tested for inhibitory activity (Fig. 4 ▸). Among them, citrate had the strongest inhibition (2 m*M*), while isocitrate did not inhibit up to a concentration of 5 m*M*; α-ketoglutarate, malate and malonate all inhibited the enzyme activity at similar concentrations (Table 3 ▸). Oxaloacetate had a limited effect. Although the affinities are weak, the inhibition was reproducible and statistically robust. Interestingly, isocitrate had no effect on *Mt*FBPaseII, eliminating the possibility of a pH-dependent effect.

The apo structure contains a citrate molecule near a proline residue that displaces the C-terminus, which is ordered in the F6P complex, forming a short β-strand. The citrate molecule is bound in one of the tetramer interfaces in a narrow, short ‘corridor’. This passage consists of Pro193 from chain *A* (Pro193*A*), His194*A* from one chain and the rings of two successive tyrosine residues (Tyr279*B* and Tyr303*B*) from the symmetry-related chain. Extending forward and backwards from this narrow passage, there is considerable polar space in the proximity of charged residues (Lys274*B*, Arg277*B*, Glu301*B* and Arg305*B*). Underneath, there is also a distal arginine residue (Arg192*B*; Fig. 2 ▸). This ligand site is occupied by the ordered C-terminus in the F6P complex and differs from that characterized in the *Ec*FBPaseI–citrate complex (PDB entry 2owz; Hines *et al.*, 2007 ▸). In the *Ec*FBPaseI–citrate complex two citrate molecules bind next to each other near a tetramer dyad, interacting with the protein surface *via* a complex network of ten hydrogen bonds near the amino-terminal helices. This citrate site can also accommodate PEP (PDB entry 2ox3), and the binding of either ligand results in enzyme activation in the presence of AMP (Hines *et al.*, 2007 ▸).

**Figure 5:**
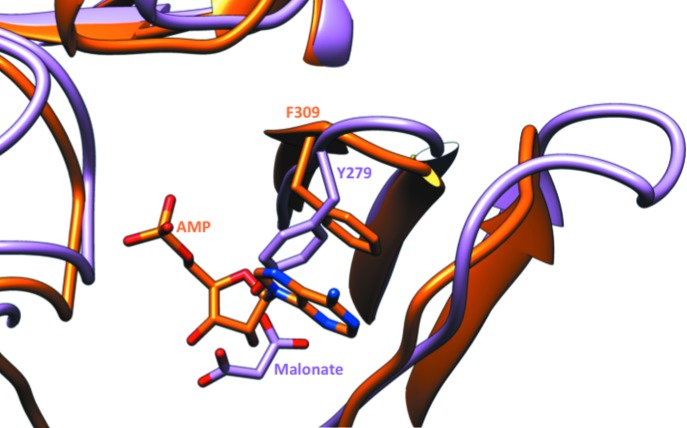
Allosteric pocket of FPBaseII. *Synechocystis* FBP/SBPase (orange) has an allosteric site with AMP bound. T84S *Mt*FBPaseII (light purple) in complex with F6P has malonate bound in the same pocket, but the amino-acid residues are not exclusively conserved. Critically, Tyr279 of *Mt*FBPaseII blocks the AMP-binding site present in *Synechocystis* FBP/SBPase (PDB entry 3rpl), which contains a phenylalanine residue (Phe309) that interacts with the adenine heterocycle by ring stacking.

The location of the malonate in the product-bound structures of *Mt*FBPaseII is near that of the AMP molecule in *Synechocystis* FBP/SBPase (Fig. 5 ▸). The *Synechocystis* enzyme (orange) has an allosteric site with AMP bound. *Mt*FBPaseII (light purple) in complex with F6P has malonate bound in the same pocket. Tyr279 of *Mt*FBPaseII blocks the AMP-binding site of PDB entry 3rpl, which contains a phenylalanine residue stacking with the adenine ring. The malonate molecules are near the allosteric AMP site of PDB entry 3rpl. The O5 of malonate is within 0.9 Å of the N6 atom of AMP when the *A* chains are superimposed. In the T84S mutant, the O7 atom of the carboxylate group of malonate is 1.1 Å (0.8 Å in the T84A mutant) from the O3′ atom of the ribose moiety of AMP. While the *B*-factor values of these crystallization reagent molecules are higher than the average value (∼90–100 Å^2^ *versus* 60–70 Å^2^), the sites could still be significantly (>0.5) occupied.

### 3.8. Solvent structure within and around the tetrameric structures   

A high concentration of glycerol is used in the preparation and storage buffers (15%), and it may participate in stabilizing the enzyme. Several glycerol molecules were found in the refined structures of both the apo enzyme and the F6P complexes. In the apo structure, glycerol molecules are found near the cleavable phosphate group in chain *A* around the periphery of the protein and two molecules are found at the dimer interface. The T84S mutant also has a glycerol molecule in the active site of chain *A* near F6P. Several glycerol molecules were found to be bound in similar locations in all structures. One such glycerol molecule is found in all chains, hydrogen-bonded to Ala132 O and Asn116 OD1. A second glycerol molecule was found in all four chains, hydrogen-bonded to Thr296 OG1, Arg291 NH2 and Arg291 NE. The average *B* factor for the discrete glycerol molecules was approximately 80 Å^2^. Even though *Mt*FBPaseII is essential for gluconeogenesis in *Mtb* in the presence of glycerol, none of the discrete glycerol molecules found appeared to be bound in any specific pocket that could indicate a regulatory or catalytic role as found in glycerol kinase (PDB entry 3h3n; Yeh *et al.*, 2009 ▸), the second enzyme of the *glpFKX* operon.

158 water molecules were found in the apo structure, 302 in the T84A mutant and 163 in the T84S mutant. The *B*-factor values of the water molecules (78, 65 and 64 Å^2^) were higher when compared with those for the overall protein structure: 68, 59 and 58 for the apo structure, the T84A mutant and the T84S mutant, respectively (Table 1 ▸).

## 4. Discussion   

The structure of the *Mt*FBPaseII protein encoded by the *glpX* gene of *Mtb* presented in this work is very similar to that of the corresponding *Ec*FBPaseII, confirming the GlpX-like phosphatase fold of this carbohydrate-phosphatase superfamily. The noncrystallographic dimer found in the structures of the *Mtb* enzyme (apo and T84A and T84S mutants) differs from the elongated dimer described for the *E. coli* enzyme. This observation, combined with the crystallographic symmetry in the two different lattices, supports the conclusion that FBPaseII is a 222 tetramer in solution. This inference is also consistent with the initial biochemical characterization of *Mt*FBPaseII (Gutka, Rukseree *et al.*, 2011 ▸), in which an oligomer (most likely a tetramer) was proposed based on molecular-weight estimation using SEC (127 kDa). The structures of the two other class II FBPases discussed (FBP/SBPases from *Synechocystis* and *T. elongatus*), which are closer homologs than *Ec*FBPaseII, are also tetramers. The *Synechocystis* enzyme structure has a tetramer in the asymmetric unit (space group *P*6_5_). A 222 homotetrameric structure is proposed as the functional unit for this enzyme class.

Comparing the catalytic residues of *Ec*FBPaseII and *Mt*FBPaseII, we find 80% conservation between the two proteins. Asp27, Glu51, Asp79, Asp82, Thr84, Tyr114, Arg159, Arg161, Asp183 and Glu208 of *Mt*FBPaseII constitute the catalytic site, corresponding to Asp33, Glu57, Asp85, Glu88, Thr90, Tyr119, Lys164, Arg166, Asp188 and Glu213 in the active site of *Ec*FBPaseII. Only Asp82 and Arg159 in *Mt*FBPaseII are not identical to their corresponding counterparts in *Ec*FBPaseII (Glu88 and Lys164), but are likely to be functionally equivalent. Based on the high level of sequence identity and functional conservation, it can be expected that the catalytic mechanism of *Mt*FBPaseII would be similar to that described for *Ec*FBPaseII (Brown *et al.*, 2009 ▸). Like the *Ec*FBPaseII structure, the *Mt*FBPaseII–F6P complex structures show convincing electron density for the metal ion (Mg^2+^) present in the active site near the cleavable phosphate group. The other active-site metals present in *Ec*FBPaseII are not present in the *Mt*FBPaseII structures, likely owing to the absence of the second phosphate group.

Arg159, Arg161 and Tyr114 form a triad that interacts with the 6-phosphate group of F6P. This is a characteristic feature among FBPases, suggesting that such an architecture determines the substrate specificity for FBP. Additionally, an Mg^2+^ ion also coordinates to the 6-phosphate group at this site, which was only found in apo *Mt*FBPaseII. The three F6P hydroxyl groups interact with three Asp side chains: 4-OH–Asp183, 3-OH–Asp82 and 2-OH–Asp181 (Fig. 3 ▸).

**Figure 6:**
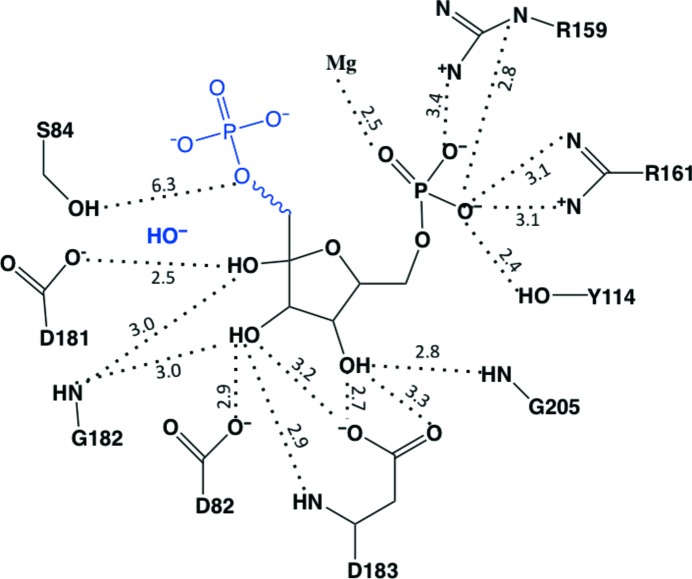
Schematic representation of the active site of the T84S *Mt*FBPaseII structure. The distances of the atoms (Å) found in the T84S *Mt*FBPaseII structure are consistent with the mechanism proposed for *Ec*FBPaseII. The cleaved phosphate and nucleophilic hydroxyl have been added in blue. A possible water has been added bridging the distance between Ser84 and the added phosphate group. This figure was prepared with *ChemDraw*.

The mechanism of FBPases has been discussed in numerous publications, with the process described as an associative two- or three-metal mechanism (Patel, Martínez-Ripoll *et al.*, 2002 ▸; Johnson *et al.*, 2001 ▸; Nishimasu *et al.*, 2004 ▸; Patel, Yenush *et al.*, 2002 ▸). The associative mechanism is initiated by deproton­ation of a water, followed by inline nucleophilic attack of the hydroxyl ion through a trigonal bipyramidal transition state, resulting in inversion of the conformation of the phosphate (Fig. 6 ▸). The dissociative mechanism describes a phosphate ester hydrolyzed through a metaphosphate intermediate. This detailed sequence of events has not been fully confirmed by corresponding crystal structures of these intermediates.

The interaction of the side chains of Thr90 (Thr84 in *Mt*FBPaseII) and Asp33 (Asp27 in *Mt*FBPaseII) in the *Ec*FBPaseII enzyme is required for catalysis. It has been suggested that this interaction increases the nucleophilicity of the Thr90 hydroxyl O atom, thereby resulting in the formation of a hydroxyl nucleophile. The hydroxyl ion subsequently attacks the phosphorus at the 1-phosphate position in FBP, generating a transition structure stabilized by the two Mg^2+^ ions in the active site. Eventually the 1-phosphate leaves, resulting in the catalytic product F6P. Since the catalytic residues involved in this mechanism and the metal ions at the active site are highly conserved between the two proteins, *Mt*FBPaseII can be assumed to follow a similar catalytic mechanism to that described for *Ec*FBPaseII.

Enzymatic characterization of the T84S variant (Bondoc *et al.*, 2017 ▸) confirmed that the enzyme was still active but was slower than the native enzyme, and it was expected to trap a reaction intermediate on growing the crystals in the presence of FBP. Our observations confirm the essentiality of the hydroxyl group, but we were unable to define the position and orientation of the T84S mutation with respect to the cleavable phosphate. The hydroxyl group of serine in the T84S variant structure is rotated compared with that of Thr90 in *Ec*­FBPaseII, perhaps explaining the lower enzyme activity. It is conceivable that the phosphate group of the substrate could orient the serine hydroxyl in the variant into an active (although sub­optimal) conformation, as suggested in the native *Ec­*FBPaseII enzyme, and retain some activity.

Comparison of the AMP allosteric site of *Synechocystis* FBP/SBPase (Feng *et al.*, 2014 ▸) with our refined structures of the two F6P complexes at high resolution provided a structural explanation for the impossibility of AMP binding at an analogous site in *Mt*FBPaseII (Fig. 5 ▸). The critical Phe309*B* residue that provides a stacking interaction with the adenine ring in the AMP-binding pocket is replaced by an inclined Tyr279 in *Mt*FBPaseII, presenting a major obstacle for AMP binding. However, our crystallographic results revealed two observations that could provide some structural support for the notion of other types of effectors for this class of enzymes.

Firstly, a citrate molecule from the crystallization medium was found at the dimer interface. The observed interactions are few and the interatomic distances are non-ideal (Fig. 2 ▸), consistent with the low affinity. Weak inhibition (IC_50_ of ∼2 m*M*) of *Mt*FBPaseII by citrate was experimentally confirmed after assaying a panel of di/tricarboxylic acids. Undoubtedly, we were able to observe this citrate molecule bound owing to its high concentration (1.0 *M*) in the crystallization medium. It is of note that isocitrate, with the additional hydroxyl group proximal to one of the carboxylates, did not have any measureable activity even at 5 m*M*. Superposition of isocitrate on the binding-pocket position of citrate suggests that the additional hydroxyl group would cause steric hindrance with the hydrophobic residues in the pocket. This citrate site differs from that found in *Ec*FBPaseI, which can accommodate citrate or PEP, resulting in activation of the enzyme at concentrations in the range 0.5–5 m*M* in the presence of 5 µ*M* AMP (Hines *et al.*, 2007 ▸).

Secondly, a malonate molecule was found in the crystal structures of the two variants complexed with F6P. These crystals did not grow in the presence of citrate, but rather in a high concentration of malonate (2.4 *M*). Molecules of malon­ate were found in the proximity of the citrate site described above. Malonate was also found to have a weak effect on the activity of the enzyme at 3–4 m*M* (Fig. 4 ▸). Given the close chemical similarity between malonate and malate, malate could occupy the site currently occupied by malonate in the F6P-bound crystal structures. Given the importance of gluconeogenesis for the survival of the *Mtb* bacterium in the latent phase, it is possible that this enzyme is regulated, as has been reported for the enzyme from *Synechocystis*.

The three refined crystal structures of *Mt*FBPaseII (native and F6P complexes) characterized here establish the quaternary structure, the monomeric fold and the active-site interactions of the only FBPase in *Mtb*. A unique citrate-binding pocket in one of the tetramer interfaces has been characterized that could be used as an initial scaffold for further chemical design. This structural information is essential for pursuing a target-based strategy for *Mtb* therapy focused on this enzyme, the gene for which has already been validated by genetic methods. Structure-based methods will be used to confirm and optimize the initial hits identified by high-throughput screening campaigns of chemical libraries. Our primary goal is to characterize and design lead inhibitors that will permit validation of this target using novel and specific chemical entities.

## Supplementary Material

PDB reference: *Mt*FBPaseII, apo, 6ayy

PDB reference: T84A mutant, complex with F6P, 6ayv

PDB reference: T84S mutant, complex with F6P, 6ayu

Supplementary Tables and Figures.. DOI: 10.1107/S2059798318002838/dw5188sup1.pdf

## Figures and Tables

**Table 1:** Data-collection and refinement statistics Values in parentheses are for the highest resolution shell.

	Apo (PDB entry 6ayy)	T84A (PDB entry 6ayv)	T84S (PDB entry 6ayu)
Wavelength (Å)	0.97856	0.97856	0.97872
Resolution range (Å)	19.68–2.60 (2.69–2.60)	38.77–2.30 (2.38–2.30)	37.78–2.20 (2.28–2.20)
Space group	*P*6_1_22	*P*6_1_22	*P*6_1_22
Unit-cell parameters (Å, °)	*a* = *b* = 131.54, *c* = 144.35, α = β = 90, γ = 120	*a* = *b* = 131.24, *c* = 144.15, α = β = 90, γ = 120	*a* = *b* = 130.89, *c* = 142.85, α = β = 90, γ = 120
Total reflections	481839	1042914	406039
Unique reflections	23145 (2254)	33106 (3247)	37186 (3651)
Multiplicity	21.6 (22.1)	23.7 (21.4)	10.8 (10.6)
Completeness (%)	99.6 (99.6)	99.9 (100.0)	100.0 (100.0)
Mean *I*/σ(*I*)	44.8 (1.7)	55.0 (2.1)	35.7 (2.1)
Wilson *B* factor (Å^2^)	62.1	49.5	49.2
*R*_merge_ †	0.079 (1.806)	0.125 (2.314)	0.082 (1.326)
*R*_p.i.m._	0.017 (0.456)	0.026 (0.469)	0.025 (0.426)
CC_1/2_ (%)	95.2 (67.2)	97.4 (69.1)	94.8 (90.7)
Reflections used in refinement	23137 (2254)	33102 (3247)	37185 (3651)
Reflections used for *R*_free_	1116 (112)	1674 (137)	1831 (193)
*R*_work_ ‡	0.211 (0.269)	0.190 (0.235)	0.224 (0.281)
*R*_free_ §	0.265 (0.349)	0.247 (0.286)	0.258 (0.304)
No. of non-H atoms	4558	5079	4626
Root-mean-square deviations			
Bond lengths (Å)	0.009	0.009	0.008
Bond angles (Å)	1.27	1.30	1.30
Ramachandran plot			
Favored (%)	94.6	96.8	96.1
Allowed (%)	4.9	3.0	3.6
Outliers (%)	0.5	0.2	0.3
Rotamer outliers (%)	0.0	0.6	0.2
Clashscore	11.0	10.6	8.7
Average *B* factor (Å^2^)	68.3	58.5	58.2

† *R*_merge_ = 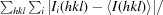 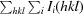, where 〈*I*(*hkl*)〉 is the mean *I*(*hkl*) value for all symmetry-related observations of that reflection.

‡ *R*_work_ = 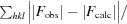 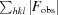, where *F*_obs_ and *F*_calc_ are the observed and calculated structure-factor amplitudes, respectively.

§ *R*_free_ is calculated using 5% of the total reflections, which were randomly selected and were not used in refinement.

**Table 2:** Structural comparisons of FBPaseII Root-mean-square deviations for C^α^ pairs from the *A* chains of wild-type *Mt*FBPaseII and variants, *Ec*FBPaseII (PDB entry 3d1r), the *Synechocystis* dual enzyme FBP/SBPase (PDB entry 3rpl) and the homologous FBP/SBPase from *T. elongatus* (PDB entry 5a5l) are presented. The upper right values are the r.m.s.d.s in Å; the lower left values are the percentages of equivalent pairs compared with the total possible pairs.

	Apo	T84A	T84S	3d1r	3rpl	5a5l
Apo	—	0.382	0.367	0.891	0.821	0.819
T84A	100	—	0.265	0.898	0.791	0.803
T84S	100	100	—	0.929	0.762	0.802
3d1r	85.9	83.4	83.7	—	0.997	1.014
3rpl	86.2	83.4	84.0	79.9	—	0.559
5a5l	86.2	83.7	83.1	83.0	96.2	—

**Table 3:** IC_50_ values of compounds against *Mt*FBPaseII Each data set was tested in triplicate and the IC_50_ value is a fit of triplicate data with the standard error reported. Isocitrate did not inhibit *Mt*FPBaseII up to 5 m*M* and an IC_50_ value could not be determined (ND).

Compound	IC_50_ (m*M*)
Citrate	2.0 ± 0.1
Isocitrate	ND
Oxaloacetate	6.4 ± 0.5
α-Ketoglutarate	3.1 ± 0.2
Malate	3.3 ± 0.1
Malonate	3.2 ± 0.1
