# Supplementary material for: Structures of the Mycobacterium tuberculosis GlpX protein (class II fructose-1,6-bisphosphatase): implications for the active oligomeric state, catalytic mechanism and citrate inhibition
Source: Acta Crystallogr D Struct Biol. 2018 Apr 3;74(Pt 4):321–31. doi: 10.1107/S2059798318002838 (PMC5892879; doi:10.1107/S2059798318002838)
Supplement: Supplementary file 1 [file d-74-00321-sup1.pdf]

STRUCTURAL
BIOLOGY

Volume 74 (2018)

Supporting information for article:

Structures of the *Mycobacterium tuberculosis* GlpX protein (class II fructose-1,6-bisphosphatase): implications for the active oligomeric state, catalytic mechanism and citrate inhibition

Nina M. Wolf, Hiten J. Gutka, Farahnaz Movahedzadeh and Celerino Abad-Zapatero

Figure S1 Boundaries of β-strands in the different β-sheets present in Class I and Class II FBPases. Definition of the β-sheets (A, B) and the boundaries of the component β-strands in the structures of previously published Class I FBPase from pig kidney (4fbp, Table S1*a*) and *E.coli* (2q8m, Table S1*b*), compared with the *E. coli* Class II FBPase (3d1r, Table S1*c*). The overall topologies of the corresponding domain structures are presented in Figs. S1(*a*), S1(*b*) and S1(*c*) respectively (domains 1, 2 in red and blue respectively). Tables and figures were prepared with PDBSum (www.ebi.ac.uk/thornton-srv/databases/pdbsum/).

Table S1(*a*). 4fbp Pig kidney (*Sus scrofa*) (Class I)

No.	Start	End	Sheet	No. resid	Edge	Sequence
1.	Thr91	Thr96	A	6	No	TCVLVT
2.	Ile103	Ile104	A	2	Yes	II
3.	Arg110	Asp121	A	12	No	RGKYVVCFDPLD
4.	Ile132	Arg140	A	9	No	IGTIFGIYR
5.	Ala161	Tyr167	A	7	No	AAGYALY
6.	Thr171	Met177	A	7	No	TMLVLAM
7.	Gly180	Asp187	A	8	No	GVNCFMLD
8.	Glu192	Arg198	A	7	Yes	EFILVDR
9.	Ile208	Ser210	B	3	No	IYS
10.	Gly241	Ala242	B	2	Yes	GA
11.	Ile261	Tyr264	B	4	No	IFMY
12.	Leu294	Thr296	B	3	Yes	LAT
13.	Ile316	Gly319	B	4	No	IILG

Figure S1(*a*)

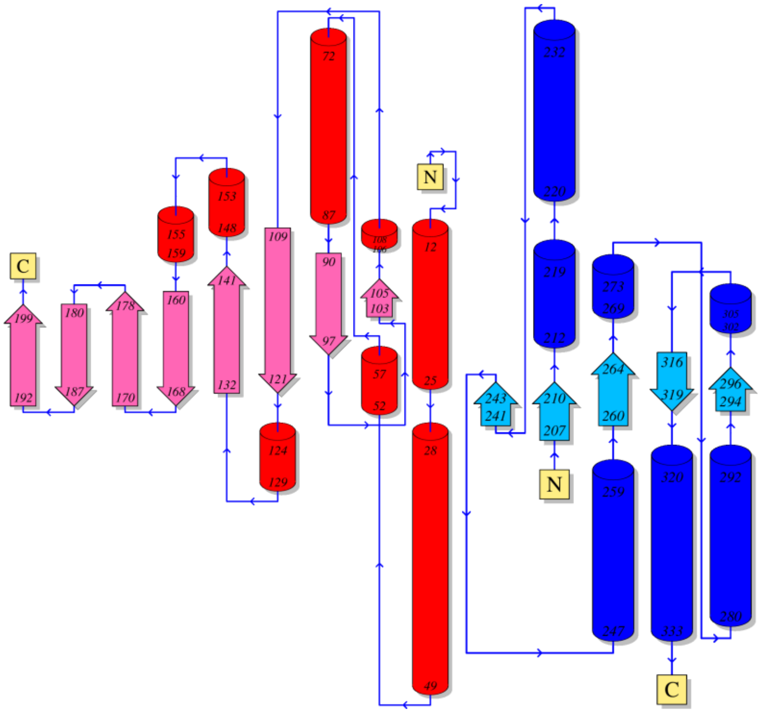

Table S1(*b*). 2q8m (*E. coli*) Class I

No.	Start	End	Sheet	No. resid	Edge	Sequence
1.	Gly85	Ser88	A	4	No	GIAS
2.	Val95	Val96	A	2	Yes	VV
3.	Tyr105	Asp113	A	9	No	YVVL-DPLD
4.	Val124	Arg132	A	9	No	VGTIFSIYR
5.	Ala155	Tyr161	A	7	No	AAGYVVY
6.	Thr165	Thr170	A	6	No	T-LVYT
7.	Val175	Tyr180	A	6	No	VHAFTY
8.	Phe187	Unk194	A	8	Yes	FCLCQER-
9.	Thr202	Ser204	B	3	No	TYS
10.	Thr236	Ser237	B	2	Yes	TS
11.	Ile256	Tyr259	B	4	No	IYLY
12.	Lys289	Ser291	B	3	Yes	KAS
13.	Phe311	Gly314	B	4	No	FFVG

Figure S1(*b*)

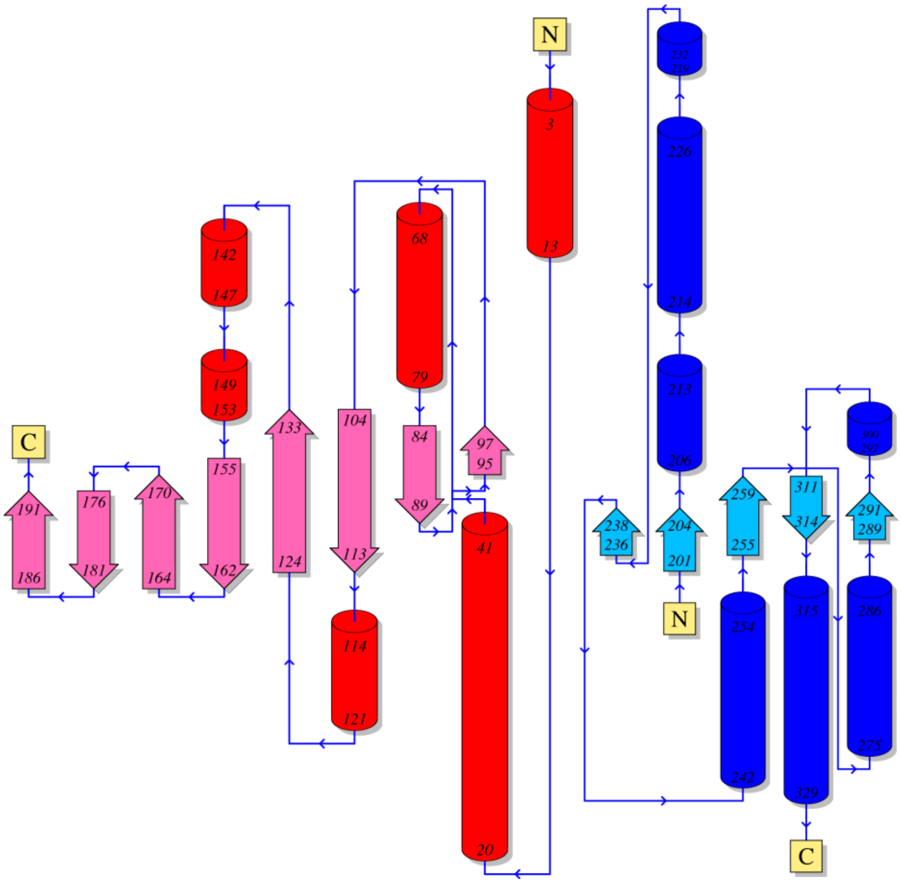
N
3
13
226
214
213
206
68
79
142
147
149
153
C
191
186
176
181
170
164
155
162
133
124
104
113
114
121
84
89
97
95
41
20
238
236
204
201
N
259
255
254
242
311
314
315
329
C
291
289
286
275

Table S1(*c*). 3d1r (*E. coli*) (Class II)

No.	Start	End	Sheet	No. resid	Edge	Sequence
1.	Asp50	Ile55	A	6	No	DGTIVI
2.	Lys71	Val72	A	2	Yes	KV
3.	Ala79	Glu88	A	10	No	AVDIAVDPIE
4.	Leu101	Asp108	A	8	No	LAVLAVGD
5.	Tyr119	Val125	B	7	No	YMEKLIV
6.	Thr158	Leu162	B	5	No	TVTIL
7.	Arg180	Ile184	B	5	Yes	RVFAI
8.	Val204	Gly210	B	7	No	VLYGIGG
9.	Asp227	Leu233	B	7	No	DMNGRLL
10.	Val266	Arg268	B	3	Yes	VLR
11.	Val278	Gly284	A	7	No	VIFSATG
12.	Ser295	Lys297	A	3	Yes	SRK
13.	Ile300	Arg309	A	10	No	IATTETLLIR
14.	Ile315	His322	A	8	Yes	IRRIQSIH

Figure S1(*c*)

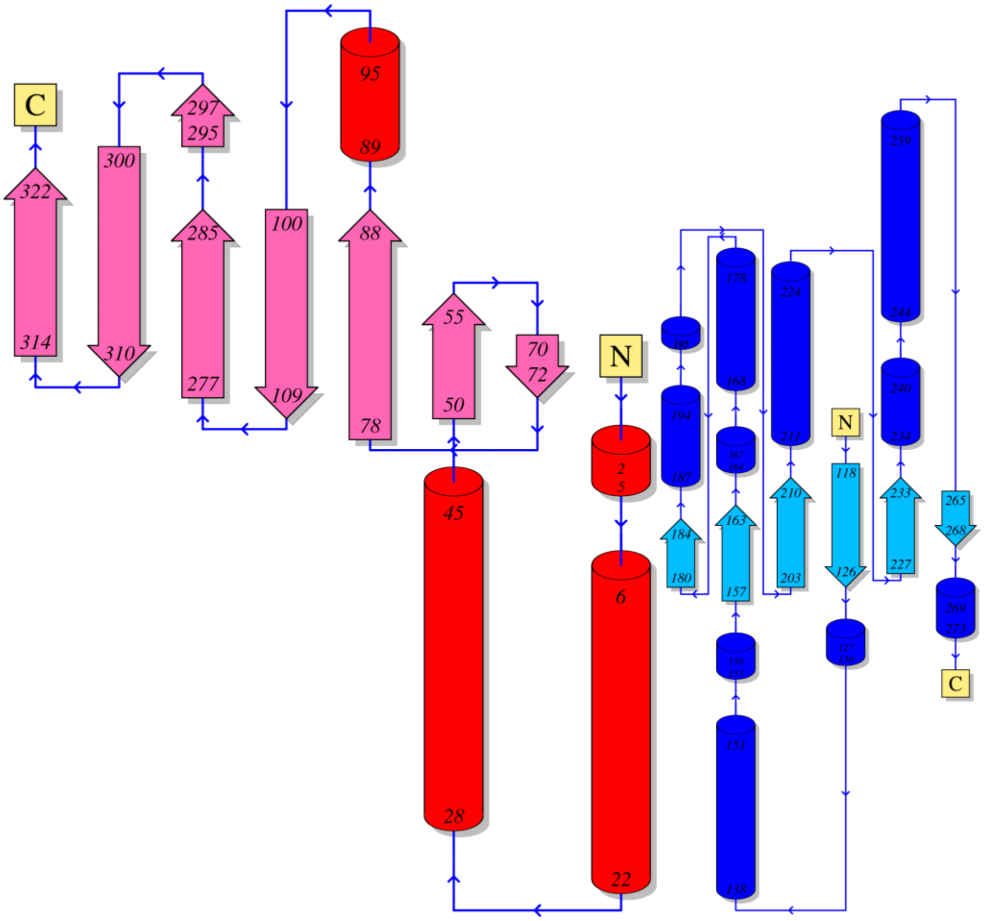
C
322
314
300
310
297
295
285
277
100
109
95
89
88
78
55
50
70
72
N
2
5
45
28
6
22
178
168
224
211
259
244
240
234
194
187
184
180
163
157
210
203
N
118
126
233
227
265
268
151
138
C

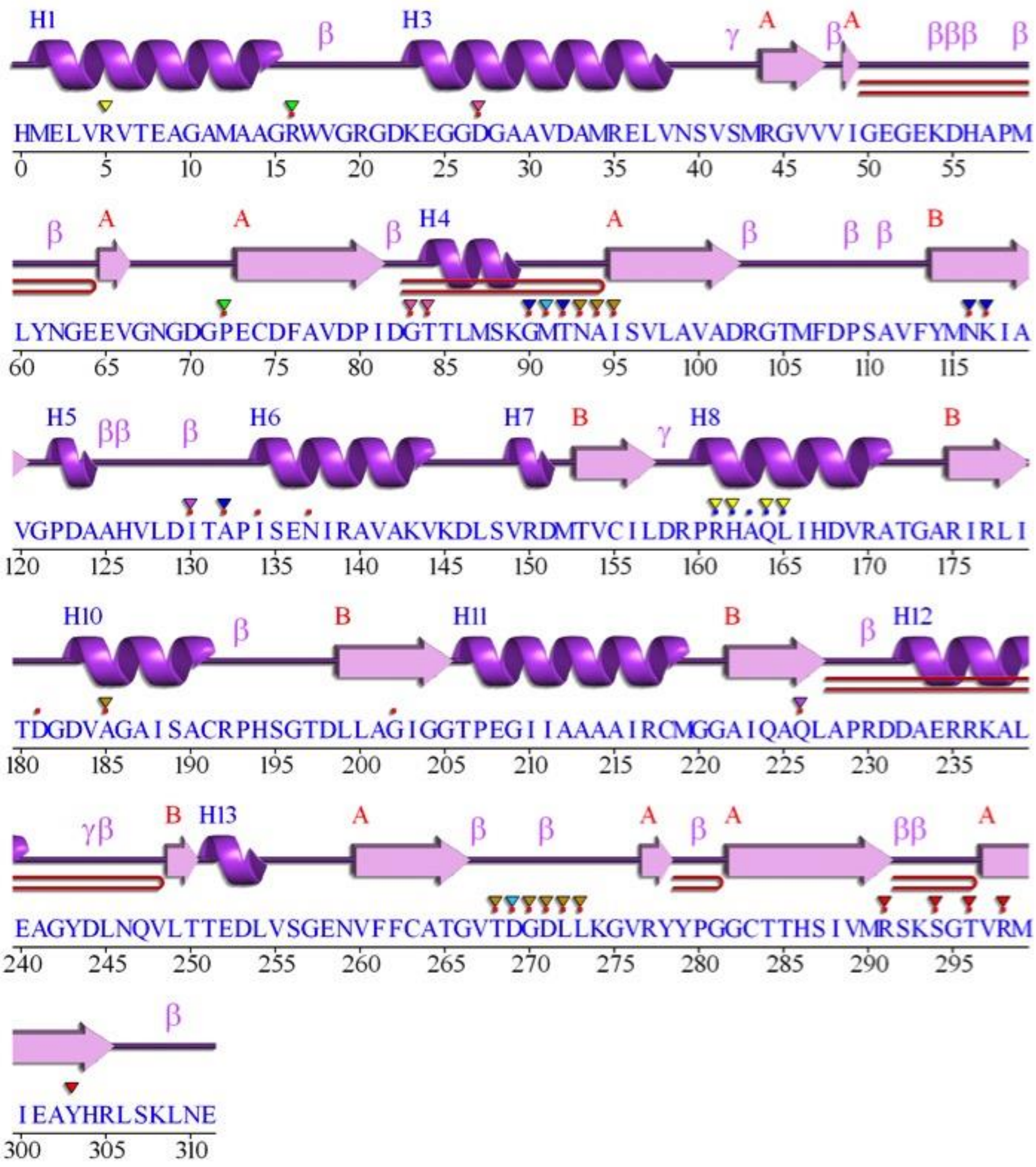

Figure S2 Secondary structure of *Mt*FBPaseII. The secondary structure for *Mt*FPBaseII is represented as α-helices (H1-H13) and β-sheets (A or B), prepared by PDBsum (Laskowski, 2001). Turns are defined as eitherβ or γ. Red squares indicated residue contacts to a ligand and blue squares to a metal. Laskowski, R. A. (2001). *Nucleic Acids Res* **29**, 221-222.

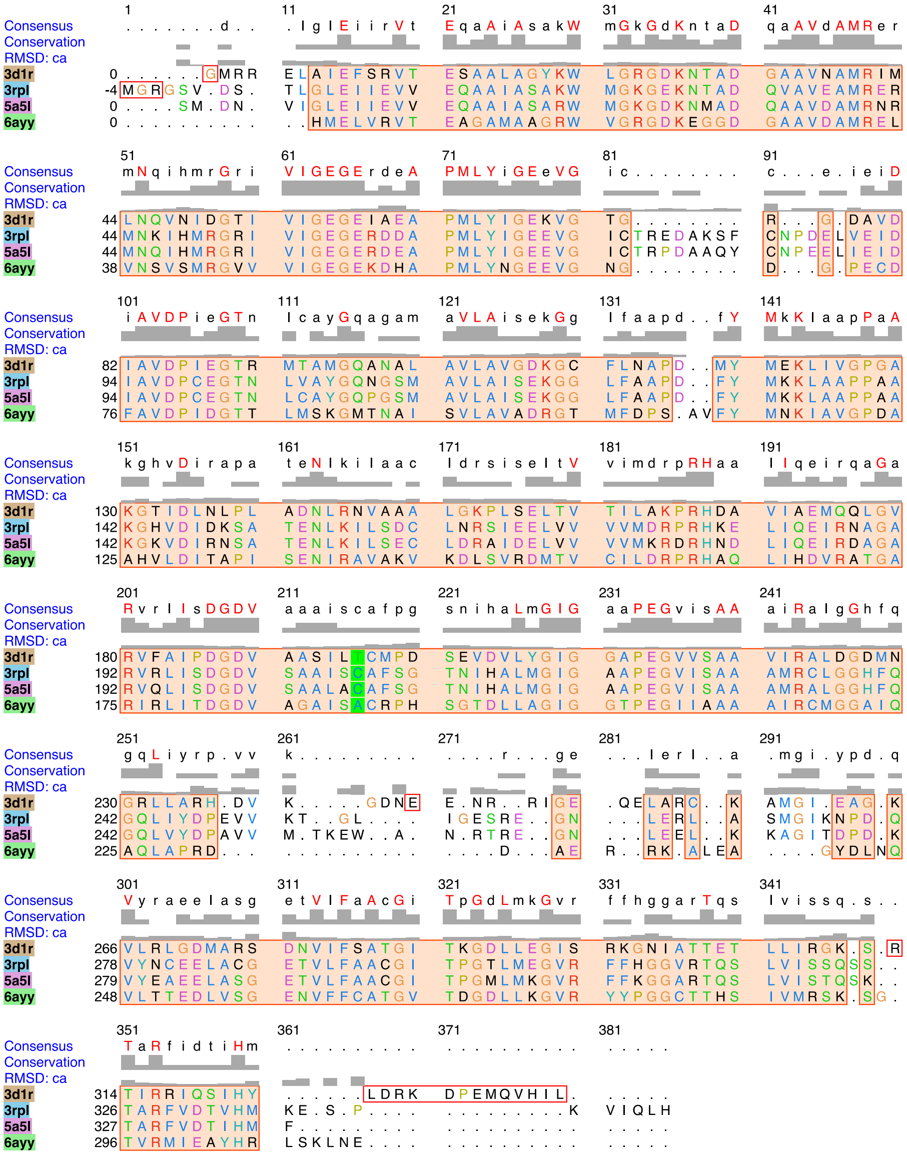

Figure S3 Structural alignment of several Class II FBPases. Structural alignment for *Mycobacterium tuberculosis* T84S (6ayy), *Escherichia coli* Class II (3d1r), *Synechocystis* FBP/SBPase (3rpl) and *Thermosynechococcus elongatus* FBP/SBPase (5a5l). Strictly conserved residues have a red capital letter in the consensus line, conserved homologous residues are in lower case and variant residues or gaps are represented by dots. The conservation profile is higher when more species have the conserved residue(s). RMSD line represents the distance of C_α pairs. Numeric details are given in Table 2 in terms of RMSD among the different proteins.

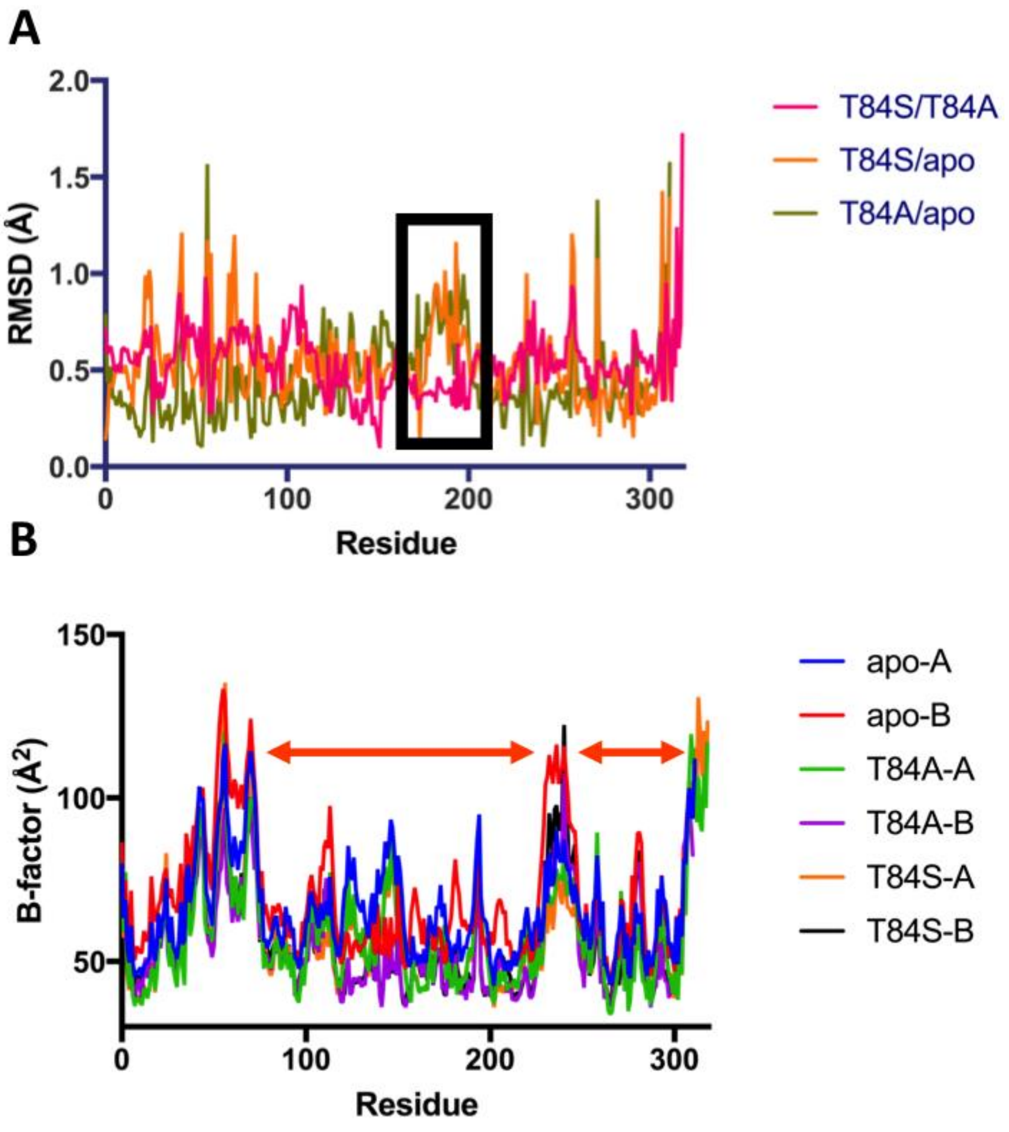

Figure S4 Comparisons of chains of *Mt*FPBaseII structures. (A) RMSD deviations among A chains from the variants and apo *Mt*FBPaseII structures. Black box highlights the region of largest structural differences between the apo and the two variants, aside from the C-terminus. (B) B-factor values for the A and B chains of *Mt*FBPaseII structures. β-sheets are bounded by double head arrows, emphasizing the most stable portions of the enzyme. Graphs were prepared with Prism.
